# MOF-Like 3D Graphene-Based Catalytic Membrane Fabricated by One-Step Laser Scribing for Robust Water Purification and Green Energy Production

**DOI:** 10.1007/s40820-022-00923-4

**Published:** 2022-08-23

**Authors:** Xinyu Huang, Liheng Li, Shuaifei Zhao, Lei Tong, Zheng Li, Zhuiri Peng, Runfeng Lin, Li Zhou, Chang Peng, Kan-Hao Xue, Lijuan Chen, Gary J. Cheng, Zhu Xiong, Lei Ye

**Affiliations:** 1 School of Optical and Electronic Information and Wuhan National Laboratory for Optoelectronics, Huazhong University of Science and Technology, Wuhan, 430074 People’s Republic of China; 2 Hubei Yangtze Memory Laboratories, Wuhan, 430205 People’s Republic of China; 3 Institute for Frontier Materials, Deakin University, Geelong, VIC 3216 Australia; 4 Key Laboratory of New Processing Technology for Nonferrous Metal and Materials (Ministry of Education), Guangxi Key Laboratory of Optical and Electronic Materials and Devices, College of Materials Science and Engineering, Guilin University of Technology, Guilin, 541004 People’s Republic of China; 5 College of Chemistry and Materials Science, Hunan Agricultural University, Hunan, 410128 People’s Republic of China; 6 School of Material Science and Engineering, Hunan University of Science and Technology, Xiangtan, Hunan Province People’s Republic of China; 7 School of Industrial Engineering and Birck Nanotechnology Centre, Purdue University, West Lafayette, IN 47907 USA; 8 Institute of Environmental Research at Greater Bay, Key Laboratory for Water Quality and Conservation of the Pearl River Delta, Ministry of Education, Guangzhou University, Guangzhou, 510006 Guangdong People’s Republic of China

**Keywords:** 3D graphene, Laser scribing, Catalytic membrane, Water purification, Hydrogen production

## Abstract

Increasing both clean water and green energy demands for survival and development are the grand challenges of our age. Here, we successfully fabricate a novel multifunctional 3D graphene-based catalytic membrane (3D-GCM) with active metal nanoparticles (AMNs) loading for simultaneously obtaining the water purification and clean energy generation, via a “green” one-step laser scribing technology. The as-prepared 3D-GCM shows high porosity and uniform distribution with AMNs, which exhibits high permeated fluxes (over 100 L m^−2^ h^−1^) and versatile super-adsorption capacities for the removal of tricky organic pollutants from wastewater under ultra-low pressure-driving (0.1 bar). After adsorption saturating, the AMNs in 3D-GCM actuates the advanced oxidization process to self-clean the fouled membrane via the catalysis, and restores the adsorption capacity well for the next time membrane separation. Most importantly, the 3D-GCM with the welding of laser scribing overcomes the lateral shear force damaging during the long-term separation. Moreover, the 3D-GCM could emit plentiful of hot electrons from AMNs under light irradiation, realizing the membrane catalytic hydrolysis reactions for hydrogen energy generation. This “green” precision manufacturing with laser scribing technology provides a feasible technology to fabricate high-efficient and robust 3D-GCM microreactor in the tricky wastewater purification and sustainable clean energy production as well.

**Supplementary Information:**

The online version contains supplementary material available at 10.1007/s40820-022-00923-4.

## Highlights

- A novel multifunctional three-dimensional graphene-metal frame film for the combination of water purification and hydrolysis hydrogen production, which has excellent purification efficiency and extremely low energy consumption.
- A “green” one-step laser scribing technology without any organic solvent in the whole preparation process.
- Metal nanoparticles as the active site can be loaded in a graphene film and wrapped by graphene, preventing undesirable aggregation and increasing catalytic active sites.

**Supplementary Information:**

The online version contains supplementary material available at 10.1007/s40820-022-00923-4.

## Graphical abstract

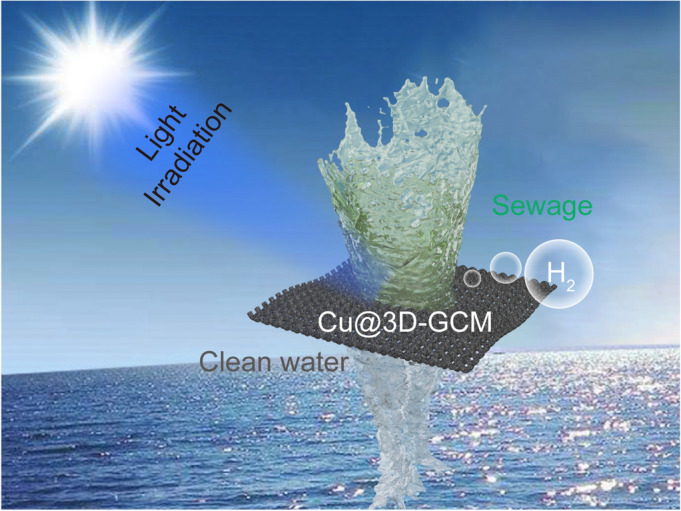

## Introduction

The ever-increasing energy and clean water demands are two major issues that currently bother many countries [1, 2]. To resolve them, this has led to extensive research on contaminated water purification as well as hydrolysis of hydrogen production [1–5], which often refers to the advanced functional nanomaterials. Catalytic membrane has both filtration and catalytic properties, and is one of the potential materials for sewage treatment and hydrogen production [2–4]. Among them, material scientists have paid the much attention to the graphene oxide (GO) membranes because of their high structure stability, exceptional water permeation and molecular sieving properties [5]. Regarding these advantages, GO nanosheets have acted as the “bricks” to construct the stacked 3D porous membranes for fast and efficiently producing the clean water from the wastewater and saline water [5, 6]. Unlike traditional polymeric membrane, the 3D graphene-based membranes (3D-GMs) enables ultrafast transport of water through defects or nanochannels between individual GO nanosheets; and in the meantime, their narrow interlayer spacings and large special surface areas provide with the high removal performances of various organics and soluble metal ions via the rejection or adsorption [5–7]. The 3D-GMs shows the better ability in breaking through the tradeoff between permeation fluxes and intercept precision while comparing with the traditional membranes [8–10]. However, traditional 3D-GMs are prepared from the GO dispersion with mass fraction less than 1.0 wt.% via the solution-processed self-assembly, electric field assistance, and vapor deposition which often costs much time and effort to stack the GO nanosheets.

Moreover, such GO membranes suffer from low stability in aqueous medium [11–13]. When the GO membranes are immersed in aqueous media and subjected to certain hydraulic pressures, polar water easily enters, swells, and separates GO nanosheets from each other, leading to delamination of GO membranes within a few hours [11–13]. Especially in practical separation system, the powerful cross-flow shearing could severely damage the GO membranes within a very short time [12]. Although a number of strategies, such as cross-linking by chemical cross-linkers or multivalent metal cations [14, 15], hydroiodic acid or hydrazine reduction [16, 17], and insertion of polyelectrolytes [18] or epoxy resins [19] have been proposed to improve stability of GO membrane, those modification avenues need vast solvents again to disperse those modifiers for the cross-linking reaction in the narrow interlayer spacings of GO nanosheets. This shows that whether engineering the architecture of 3D-GMs or the cross-linking modification of 3D-GMs either cause additional environmental risks due to the large amount of chemicals or waste solvents involved. Unfortunately, there is no better choice in using the solution self-assembly to fabricate the advanced 3D-GMs, let alone modify or functionalize 3D-GMs. For example, researchers recently focus on engineering the 3D graphene-based catalytic membranes (3D-GCMs) via the incorporating of the active metal nanoparticles (MNPs) into membrane pores [20, 21]. Based on the encapsulated MNPs, the 3D-GCMs exhibit the various state-of-the-art properties such as the clean energy production of H_2_ [21]. In order to enhance the production efficiency of H_2_, the plasmonic photocatalysis is widely implemented to cause the hot-electrons generation, which accelerates the catalytic dissociation of water at the surface of MNPs [22, 23]. Meanwhile, the electron transfer at the surface of MNPs could also activate many kinds of peroxides for the generation of reactive oxygen radicals (ROS), which displays the strong oxidation in the degradation of recalcitrant organic pollutants (organic dyes and antibiotics) in water [20].

However, the intrinsic ultrafast recombination for photoinduced electron–hole pairs in the metal nanoparticle can hinder the hot-electron transfer to the outside. The phenomenon commonly results from large diameter of nanoparticles, and hence reduces the efficiency of plasmonic photocatalysis [24]. To maximize the separation of electron–hole pairs, it is essential for the MNPs to effectively prevent undesirable aggregation and couple to effective electron acceptors with high electron mobility [25]. For obtaining the excellent photocatalytic 3D-GCMs, the precursors of MNPs are usually premixed with GO dispersion [20, 23–25]. By virtue of the functional groups of GO nanosheets, the MNP precursors are evenly anchored on the GO surfaces, and then orderly undergo the crystal growing, reduction and solution self-assembly, ultimately realizing the preparation of 3D-GCMs [20, 21, 23]. This fabrication process is time-costing, solvent and chemical agents wasting as well as the metal catalysts. To this end, how to pursue “green” synthesis approach plays vital roles in shaping the structural property of the scalable 3D-GCMs for application that involves hydrogen production and water purification.

**Fig. 1:**
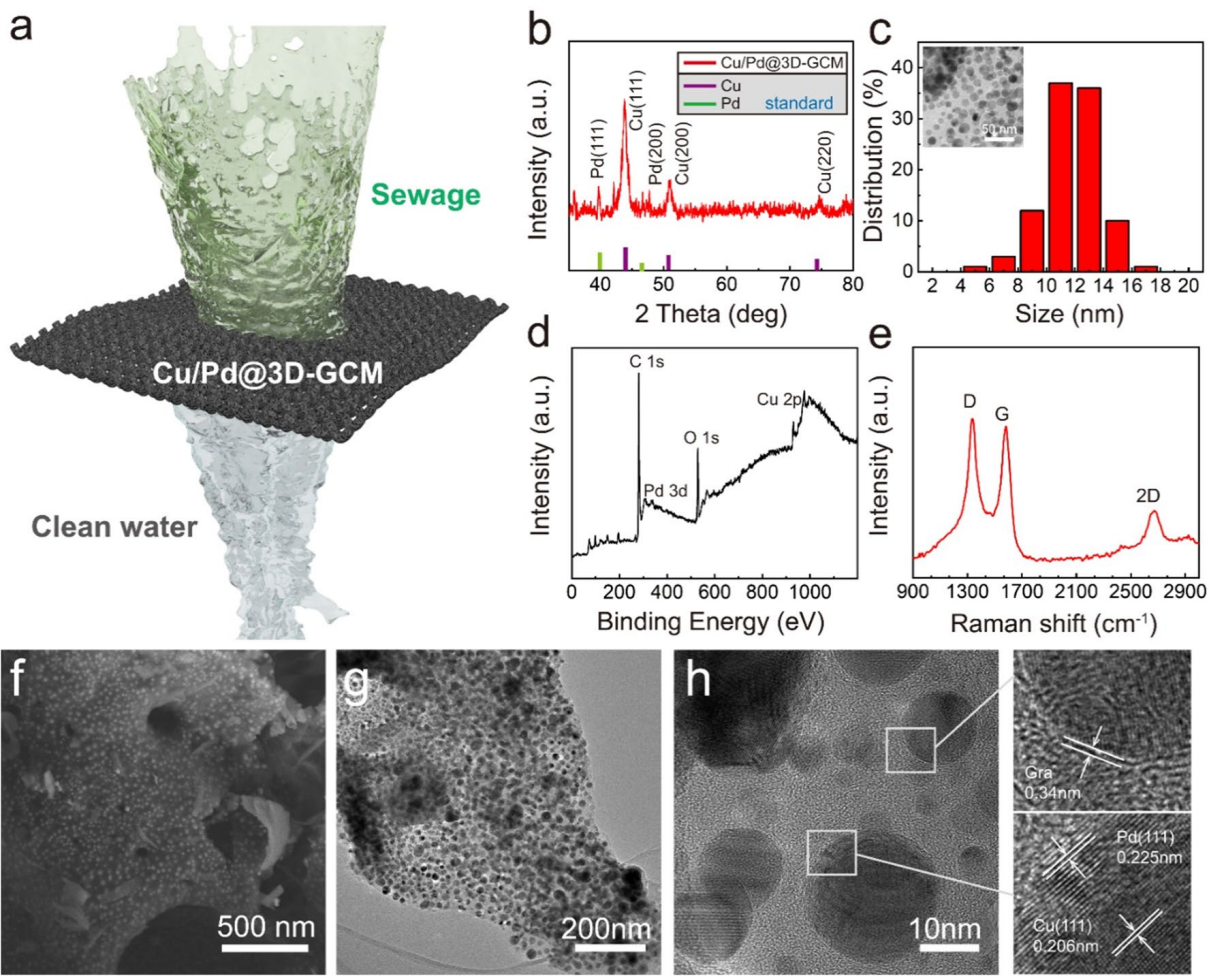
**a** Schematic diagram of the sewage treatment in Cu/Pd@3D-GCM. **b** XRD patterns, **c** Size distribution of MNPs, **d** The XPS spectra of Cu/Pd@3D-GCM, and **e** Raman spectra of Cu/Pd@3D-GCM. **f** SEM image, **g** TEM image, and **h** high-resolution TEM images of Cu/Pd@3D-GCM

Herein, we introduce a green, cost-effective, and ultrafast strategy to design the robust 3D-GCMs via the advanced laser scribing technology [26, 27]. The ultrafast heating of the GO generates endogenous pyrolysis, which not only welds the GO nanosheets for engineering the robust 3D-GM, but also in situ sinters the MNPs on the GO nanosheet surfaces. The clever intercalation of MNPs into graphene sheets can prevent the aggregation of noble MNPs and enhances efficiency of the electron transfer. The green fabrication process does not require any solution or chemical reagents. More interestingly, the microstructure of 3D-GCMs is like the porous metal–organic frameworks (MOFs) with high porosity, strong mechanical, large surface area after the advanced laser scribing engineering. Correspondingly, our novel 3D-GCM with excellent dynamic adsorption capacity can remove various trace contaminants, generating a large amount of cleaning water with a very low hydraulic pressure and in a short time, which greatly reduces the energy consumption and improves the efficiency (Fig. 1a). The loaded active MNPs can also realize the self-cleaning of 3D-GCM through an advanced oxidation process (AOP) [20]. Besides, non-radiative surface plasmon decay caused by surface plasmon resonance of MNPs involve the generation of hot electrons, which continuously generate hydrogen under ultraviolet light radiation. Therefore, our work provides an efficient, cost-effective, and green technology for the development of novel membranes for industrial applications that require advanced wastewater purification and energy regeneration.

## Experimental Sections

### Preparation of 3D-GCM

MOF powders fixed by metal copper sheet mold was sandwiched between two slides and then laser scribing was performed on the MOF powders. A nanosecond pulsed laser (YLP, IPG photonics) was used as the strict energy source (1064 nm wavelength, 80 ns pulse width). Among them, the laser can be focused to a spot size of 50–200 μm. Any graphic can be programmed on the MOF layer by using programmatic control of the beam direction of the galvanometer (6240H, Cambridge Technology Inc.) to prepare a large-area membrane.

### Characterization

X-ray diffraction (XRD) was conducted from a PANalytical B.V. X-ray diffractometer. Field emission transmission electron microscope (FTEM, Tecnai G2 F30, Netherlands) equipped with energy dispersive spectroscopy (EDS) was used to analyze the membranes morphology. Elements on the surface of samples were characterized by X-ray photoelectron spectroscopy (XPS, AXIS-ULTRA DLD-600 W, Japan). Raman spectroscopy (LabRAM HR800, France) was performed to confirm the graphene nanocomposites. Ultraviolet–visible-near infrared Spectrophotometer (UV–Vis-NIR, SolidSpec-3700, Japan) was conducted to estimate the optical response of the as-synthesized samples. Fourier Transform Infrared Spectroscopy (FTIR) spectra was conducted by VERTEX 70 instrument (Germany). N_2_ absorption isotherms were measured using an Autosorb-IQ2 (America).

### Performance Tests of 3D-GCM

A cross-flow system was employed to evaluate the permeability of 3D-GCM under a certain hydraulic pressure. The effective area of the membrane was 3.14 cm^2^. The deionized (DI) water was used to compact the membrane to reach a steady state at 0.1 bar. After 5 h of compacting, the pure water flux was automatically recorded every 5 min for 60 min under 0.1 bar at 25 °C. To obtain the most accurate water flux through reducing the deviation, we recorded five sets of data to obtain the average value, and to calculate the water flux and the ultrafiltration coefficients (*K*_*uc*_) of Cu/Pd@3D-GCM by Eqs. (1) and (2):

1 $$ J = \frac{V}{S \times t} $$

2 $$ K_{uc} = \frac{J}{P} $$

where *V*, *S*, and *t* are the volume of the permeated (L), the area of the membrane (m^2^), and the time required for the infiltration process (h), respectively. *P* is the driving pressure.

The rejection properties of 3D-GCM were investigated using Rhodamine B (RhB, 20 ppm), as the representative foulant suitable for use in repellency and antifouling tests. A peristaltic pump (BT300-1F, Longer Pump, China) was used to regulate the flow rate. A mixed solution was flowed through the membrane under 0.1 bar. After the system is stable, the flux data were recorded once every 10 min, and the filtrate was collected. At the end of the filtration test, a sufficient amount of permeates and residue was collected and sealed separately. An Ultraviolet–visible spectrophotometer (UV–Vis, TU1810, Persee, China) was used to identify the concentration of RhB in the cumulative sample, whose absorption peak is at 554 nm. The removal efficiency in Eq. (3):

3 $$ R = \left( {1 - \frac{C}{{C_{0} }}} \right) + 100\% $$

where *R* is the soiling agent rejection (%), and *C* and *C*_*0*_ are the soiling agent concentrations (mg L^−1^) in the permeated and feed solution, respectively.

The adsorption capability of 3D-GCM:

4 $$ q = \frac{{\left( {C_{0} - C_{t} } \right)V}}{m} $$

where *C*_*0*_ (*C*_*t*_) are the initial real-time concentrations of pollutants in water during the membrane separation process, respectively. *V* is the volume of real-time feeding solution. *m* is the weight of 3D-GCM.

### Photocatalytic Degradation Performance of 3D-GCM for Organic Pollutants

For dynamic photocatalytic degradation, RhB (10 ppm) solution was added to the membrane reactor for filtration. The solution flowed through the catalytic membrane in the dark for the first 30 min to obtain saturated adsorption. The adsorption–desorption equilibrium was established in the surface of 3D-GCM. After saturation adsorption of RhB solution in the dark, the RhB solution is stopped. Then H_2_O_2_ solution (5 µL H_2_O_2_ per 10 mL water) flows through the catalytic membrane under UV light (8 W, 350 nm). The filtrate was tested for measurable UV–Vis (TU1810, Persee, China) absorption to record complete rejection and adsorption of the organic pollutant. Furthermore, after a given time interval, the color change of the feed solution under the light irradiation was observed and the UV–Vis (TU1810, Persee, China) spectrum was recorded to analyze the filtrate. The dynamic photocatalytic degradation was repeated for five times to evaluate the stability of the catalyst membrane.

### Photocatalytic Activity of 3D-GCM for Hydrogen Evolution

The hydrogen production experiment was carried out in a photocatalytic reactor at room temperature (300 K). First, argon was used to purge the reactor for 30 min. Then a 300 W xenon arc lamp with an intensity of 100 mW cm^−2^ was used for illumination. Here, 3D-GCM is suspended in 10 mL of aqueous solution, including 9 mL of DI water and 1 mL of methanol. Gas chromatography is used to analyze gas samples. In the stability test, the hydrogen evolution experiment of 3D-GCM was performed every 4 h and repeated 5 times.

## Results and Discussion

### Structure and Morphology of 3D-GCMs

MOFs possess highly ordered porous structures, diverse metal, or organic compositions, and adjustable crystal morphologies. In this work, the MOF-like 3D-GCMs were prepared directly through a one-step laser scribing process. During the preparation process, the metal ions were reduced to MNPs in 3D-GCMs with tailorable size under various laser power and pulse conditions. Meanwhile, MOF layer was designed as a precursor, with metal ions assembled from two types of ions (mass ratio of Cu and Pd, 10:1), which was fixed with a metal copper sheet mold by two glass slides. A nanosecond pulsed laser (1064 nm) with a pulse duration of 80 ns was used as a precise energy source to scan the MOF layer. This enabled scalable manufacturing of large-area filter membranes (see Supporting Information for the details of the preparation methods). After laser scribing, the metal material and GO nanosheets were converted into a continuous and mechanically strong membrane with an area of 3.14 cm^2^ and a thickness of 500 μm only limited by the Cu mold. Figure S1 illustrates the process for the preparation of metal-loaded 3D-GCM by laser scribing. Different from direct thermal treatment, MNPs wrapped with graphene can achieve the purpose of non-agglomeration and higher density distribution on the carrier.

To confirm the crystal structure of the synthesized Cu and Pd MNPs, the XRD pattern is presented in Fig. 1b. The XRD pattern of 3D-GCM exhibits three characteristic peaks at 2θ = 44.0°, 51.0°, and 74.5° corresponding to the crystalline planes of (111), (200), and (220) of Cu MNPs, respectively [28]. For the XRD peaks of Pd, two characteristic peaks at 2θ = 40.0° and 46.5°, corresponding to the crystalline planes of (111) and (220) of Pd MNPs [29]. Hence, the MNPs samples are composed of Cu and Pd. The specific surface areas and pore structures of the samples were investigated by measuring the nitrogen adsorption isotherms (Fig. S2a). Due to the high metal content, the obtained 3D-GCM shows a surface area of 50 m^2^ g^−1^. Such surface area can be attributed to the minimal damage to the material during the ultrafast laser scribing process, which benefits the adsorption for the organic pollutants. The Cu/Pd MNPs with an average particle diameter of 12.5 nm are uniformly distributed in 3D-GCM (Fig. 1c).

To further prove the reduction in metal ion, we carried out high-resolution XPS characterization. The XPS spectra of the C 1*s*, O 1*s*, Cu 2*p* and Pd 3*d* core levels for the 3D-GCM are shown in Fig. 1d. The XPS spectra shows a characteristic peak of C 1*s* at 284.3 eV and O 1*s* at 530.9 eV. Cu 2*p*_3/2_ and Cu 2*p*_1/2_ peaks of 3D-GCM are located at bonding energy of 933.0 and 952.8 eV, respectively (Fig. S2c), consistent with metallic Cu^0^ [30]. The Pd 3*d*_5/2_ and Pd 3*d*_3/2_ for Cu/Pd@3D-GCM also shows the corresponding XPS characteristic peaks, respectively [31]. The *sp*^2^ bonded carbon (C–C, 284.8 eV) at C 1*s* spectra of MNP-G confirm that the particles of MNPs coexist in 3D-GCM. Compared with the FTIR spectra of Cu-MOF in the reference [32], the decrease in intensity for O–H stretching frequency in Cu/Pd@3D-GCM (approximately 3000–3400 cm^−1^) can be attributed to the interactions between MNPs and OH groups, indicating a strong interaction between the graphene and MNPs (Fig. S2d). Furthermore, we resorted to the Raman spectra to demonstrate the quality and stability of the prepared graphene. The Raman spectra of 3D-GCM for few-layer graphene are illustrated in Fig. 1e, with peaks located at 2650, 1590, and 1325 cm^−1^, corresponding to 2D, G, and D modes, respectively [33]. The supreme structural quality of graphene has been confirmed, showing the non-negative influence of the attached MNPs. Additionally, the intensity ratio of the G and D peaks, characterizing the quantities of defects in graphene-based materials [33–35], is 1.05 under 4.5 W laser power, which confirms the formation of highly crystalline graphene with few layers.

The SEM image of 3D-GCM in Fig. 1f clearly illustrates a coralline-like morphology with a porous structure, which is different from the product obtained by traditional pyrolysis [36]. A regular arrangement of dark spherical spots, which correspond to nanoparticles with ~ 12 nm diameter, is clearly observed along with the support in the TEM micrographs of 3D-GCM (Fig. 1g). Each nanoparticle is uniformly dispersed without significant aggregation. Furthermore, the lattice fringe corresponding to the Cu (111) and Pd (111) structure observed from the high magnification TEM (Fig. 1h, inset), is coherently extended for each nanoparticle, indicating the formation of an individual crystal structure. In addition, the layer distance of 0.34 nm is observed from the high-resolution TEM image (Fig. 1h, inset), which also confirms the formation of few-layered graphene and the efficient reduction in 3D graphene. Moreover, the MNPs produced by laser scribing are uniformly distributed on the whole framework and the layered pores are clearly visible and abundant. In addition, two kinds of morphology, including coralline-like products filled with ultrafine nanoparticles and dense nanobubble-like products, can be observed (Fig. 1f), because MNPs on the surface and inside 3D-GCM were subjected to different laser intensities. Since the laser energy suffers decay in the irradiation direction, the MNPs formed on the outer surface are prone to melting and evaporation from the graphene shell under sufficient irradiation to form graphene nanobubbles, while the inner MNPs are preserved and wrapped by the graphene shell layer. The MNPs also contribute to the formation of graphene during laser scribing due to their unique catalytic effect on graphene growth [37]. Figure S3 illustrates the EDS mapping results. The color intensities indicate the uniformity and density of the MNPs consisting of mostly MNPs and C elements.

### Membrane Dynamic Adsorptive and Photo-regenerative Performance

**Fig. 2:**
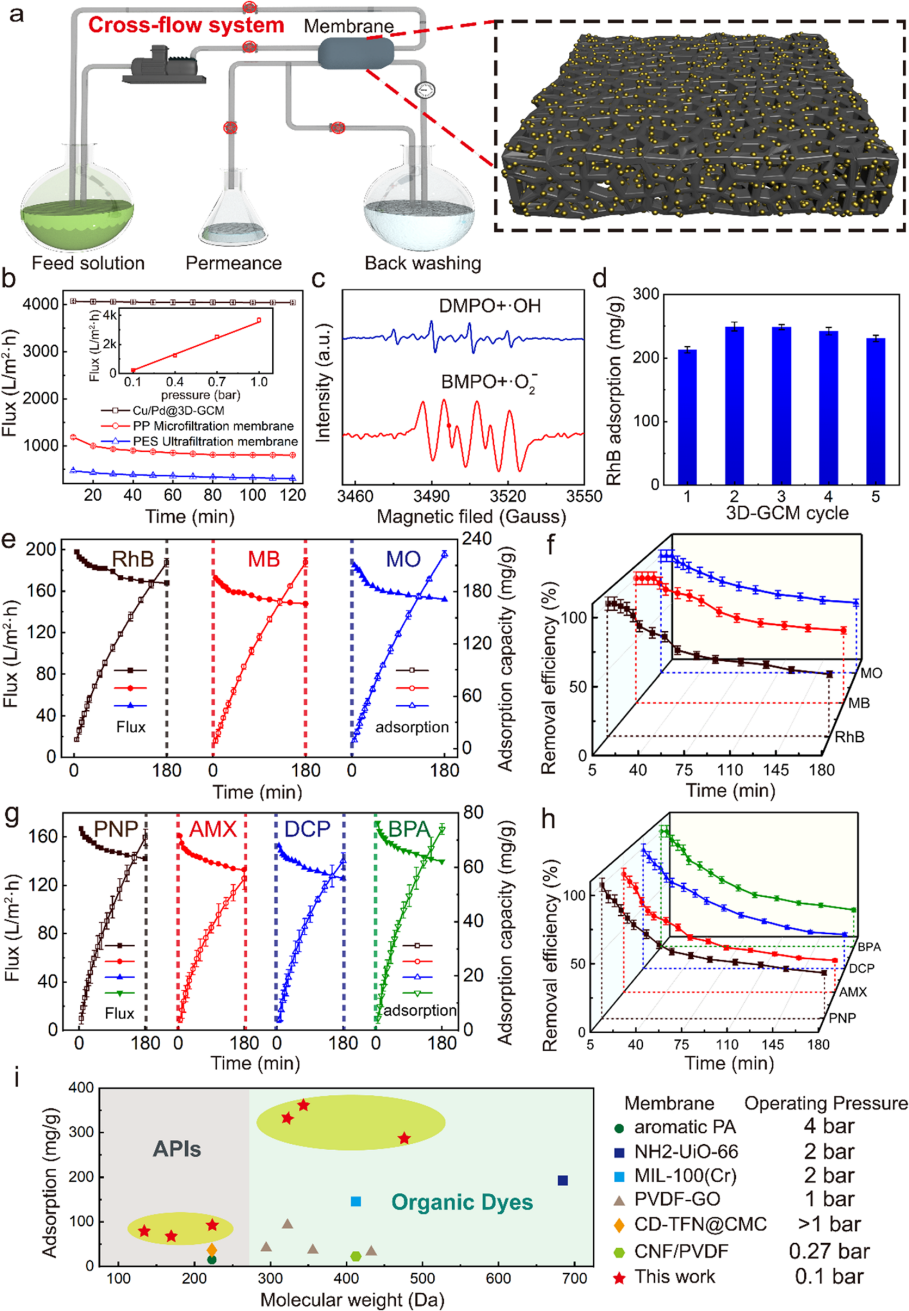
**a** Schematic diagram of the cross-flow system, inset: schematic illustration of the catalytic membrane loaded with MNPs. **b** Water fluxes of Cu/Pd@3D-GCM (black), PP microfiltration (red), and PES ultrafiltration membrane (blue). Inset: relationship between pure water flux and driving pressure for Cu/Pd@3D-GCM. **c** EPR spectra of DMPO/·OH adducts and BMPO/·O_2_^−^ adducts over 3D-GCM. **d** The adsorption capacity for RhB of the Cu/Pd@3D-GCM in the presence of H_2_O_2_ after consecutive regeneration cycles. **e–h** Time-dependent Flux, adsorption capacity, and removal efficiency of each pollutant by Cu/Pd@3D-GCM. **i** Performance comparison of 3D-GCM with other membranes reported in literature

To investigate the separation performance and antifouling performance of the 3D-GCM, a cross-flow system was employed to evaluate the permeability of Cu/Pd@3D-GCM (Fig. 2a). Figure 2b records the permeability of the pure water flux until it reaches a steady state. For our sample, it was basically unchanged after reaching a steady state (4050 L m^−2^ h^−1^ bar^−1^). Water flux relative to pressure applied on the Cu/Pd@3D-GCM is shown in the inset of Fig. 2b. The high flux (239 L m^−2^ h^−1^) under extremely low driving pressure (0.1 bar) indicates very low energy consumption in operation. The ultrafiltration coefficient (*K*_*uc*_) has been used to reflect the water permeability of the membrane. According to Eq. (2), *K*_*uc*_ reaches 4050 L m^−2^ h^−1^ bar^−1^, showing the supreme permeability at such a low driving pressure. We also tested conventional polyethersulfone (PES, 120 μm thickness, 0.1 μm average pore size, Membrana) and polypropylene (PP, 100 μm thickness, 0.22 μm average pore size, Membrana) membranes for comparison (Fig. 2b). The Cu/Pd@3D-GCM provided the highest maximum value in pure water flux compared to PES ultrafiltration and PP microfiltration membranes, as clearly demonstrated in Fig. 2b. In a typical experiment, RhB (20 ppm) was added to the cross-flow system to perform the dynamic filtration at 0.1 bar. The 100% removal rate in the first 10 min indicated complete rejection of dyes by 3D-GCM. After adsorption for 3 h, the removal rate dropped to 50%, indicating the gradual saturation of adsorption site. These results confirm the excellent separation performance of the membrane (Fig. S4). Generally, for textile wastewater treatment, the accumulation of organic pollutants leads to serious membrane fouling. The prepared Cu/Pd@3D-GCM exhibited high catalytic activity due to the existence of metal-reducing nanoparticles, which equipped the membranes with self-cleaning and antifouling capabilities. To evaluate the self-cleaning performance of Cu/Pd@3D-GCM, a dynamic measurement was performed to combine filtration and catalytic degradation into one process in the presence of H_2_O_2_ (10 μL per 50 mL water) for photocatalysis under the UV light. During the cross-flow filtration process, RhB molecules were completely retained by the nanopores of Cu/Pd@3D-GCM. Then, the adsorbed RhB molecules can be efficiently degraded. During the process, reactive oxygen-containing radicals were obtained via the activation of H_2_O_2_, which was confirmed by electron paramagnetic resonance spectroscopy (EPR) (Fig. 2c). The hydroxyl radicals were captured by spin traps 5-tert-butoxy carbonyl 5-methyl-1-pyrroline N-oxide (BMPO) and 5, 5-dimethyl N-oxide pyrroline (DMPO). Because of the hyperfine interaction between the electron spin of the free radicals and the orbital spin of N atoms in spin traps, the EPR spectra of DMPO/·OH was a 4-line spectrum with an intensity ratio of 1:2:2:1 with a spacing of 14 G in the magnetic field. Moreover, the characteristic spectrum of BMPO/·O_2_^−^ splits into 4 single lines with relative intensities of 1:1:1:1. The results indicate that ·OH and ·O_2_^−^ were simultaneously generated during the degradation process. Therefore, after four times of regeneration cycles, the dynamic RhB removal of Cu/Pd@3D-GCM could almost recover the initial value and the adsorption capacity of Cu/Pd@3D-GCM increased slightly (Figs. 2d and S4). Figure S5 shows that Cu/Pd@3D-GCM had stable removal efficiency and H_2_ evolution during 540 min under five cycles of reaction. The mechanical property of Cu/Pd@3D-GCM has the highest tensile strength value of 36.97 kPa and the highest elongation of 2.30% (Fig. S6). Synergistic results showed that the self-cleaning capability, high stability, and reproducibility of Cu/Pd@3D-GCM could minimize membrane cleaning and operating costs, showing great promise in the practical application of wastewater purification.

We investigated Cu/Pd@3D-GCM for the removal of various organic pollutants of industrial significance, including organic dyes and active pharmaceutical ingredients (APIs) with different molecular sizes and functionalities (Fig. 2e-h). Three distinct organic dyes, including RhB, methyl orange (MO), and methylene blue (MB) and four APIs, including p-nitrophenol (PNP), amoxicillin (AMX), dichlorophenol (DCP), and bisphenol A (BPA) were selected as the model aqueous contaminants for filtration test [38]. In the experiment, the removal rate for the three dyes reached 100% within 10 min. And the rejection values for APIs were more than 90%. At the same time, the slight decrease in flux showed good antifouling performance. In addition, the adsorption kinetics of different pollutants of Cu/Pd@3D-GCM regenerated multiple times are shown in Tables S1 and S2. The pseudo-first-order and pseudo-second-order models were used to describe the adsorption process and mechanism [38]. The quasi-first-order model correlation coefficient *R*^*2*^ > 0.99 indicates the rate at which external diffusion mass transfer dominated the rate of the adsorption process. Simultaneously, the curves of pseudo-second-order kinetic model also showed an ideal fit, concluding that chemical adsorption dominated the adsorption process [39]. According to the calculations above, the adsorption of organic pollutants and APIs of 3D-GCM was mainly controlled by external diffusion and chemical adsorption. Based on these experiments, 3D-GCM with satisfactory pollutant removal efficiencies could be feasible for removing other pollutants. The adsorption properties of typical graphene-based catalysts were compared (Table S3). The superior activity can be understood from the following aspects. The 3D-GCM with sufficient pores and nanoscale metal particles maximizes the surface area of the active phase. Meanwhile, we also take advantage of the dynamic catalysis, where the degradation of the contaminant mainly occurs inside the membranes to further increase the active site of the reaction. In addition, with the reduction in metal particle size and their high loading, the active components are highly dispersed and the utilization efficiency of metal particle catalyst is greatly improved. Also, the cost of 3D-GCM is a key factor for its commercialization, while the functional cost of each 3D-GCM with a diameter of 3.14 cm^2^ is roughly estimated to be less than $0.5. In addition, the operational energy consumption and removal efficiency of Cu/Pd@3D-GCM are much better compared with other membranes (Fig. 2i), indicating great potential in actual application of our membrane.

### Photocatalytic H_2_ Evolution Performance of 3D-GCM in Separation of Wastewater

**Fig. 3:**
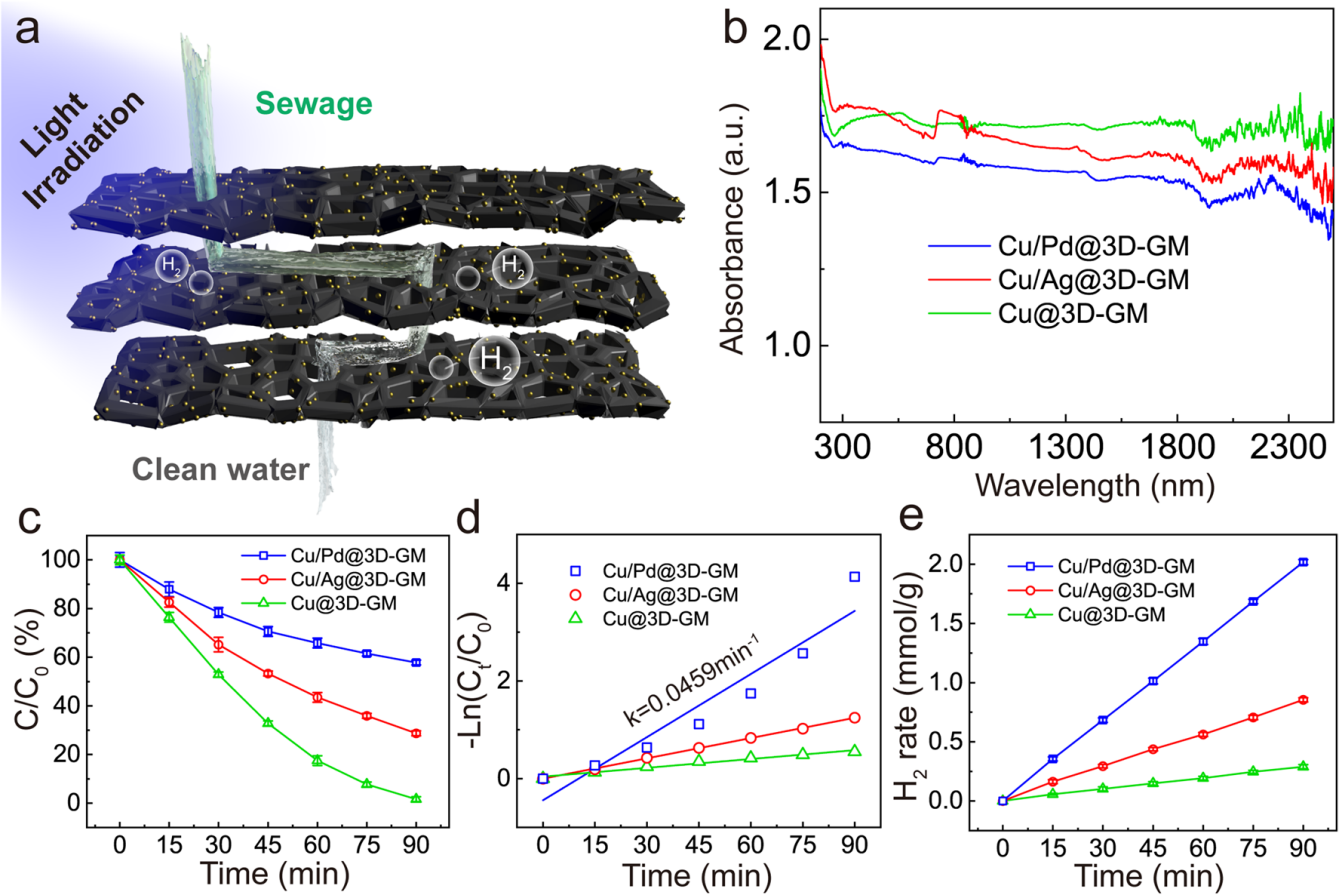
**a** Schematic diagram of the photocatalysis process in a 3D-GCM. **b** UV–Vis-NIR absorption spectra, **c, e** photocatalytic degradation behaviors and H_2_ evolution rates of three membranes (Cu@3D-GCM, Cu/Ag@3D-GCM, and Cu/Pd@3D-GCM). **d** The plot of ln(C_t_/C_0_) of RhB versus time

To demonstrate the universality of the preparation method and to confirm the photocatalytic performance based on plasmon enhancement, two other membranes anchored with Cu and Cu/Ag (5:1) alloys were successfully prepared by laser scribing, respectively. Based on the above results, the concept of 3D-GCM photocatalytic microreactors has been proposed. Figure 3a schematically illustrates the photodegradation process and photocatalytic hydrogen evolution of the microreactor. Compared with the traditional photocatalysis setup, the suspension of the catalyst often needs to be mechanically stirred [40]. Such step ensures complete mixing of catalyst and reagent. By contrast, the fabricated 3D-GCM directly guides the reactants into its open porous structures through adsorption, in close proximity to the photocatalytic sites, which simplifies the whole reaction setup. The SEM images and XRD patterns are shown in Figs. S7-S9. To estimate the optical response of the as-synthesized 3D-GCM, the UV–Vis-NIR was carried out. As shown in Fig. 3b, The UV–Vis-NIR diffuse reflectance spectra show extremely strong absorption in the region of approximately 200–2500 nm, indicating that they have considerable light absorption capacity, which most likely stems from the close packing between MNPs and 3D-GCM. In addition, Cu/Ag@3D-GCM and Cu/Pd@3D-GCM exhibit more enhanced absorption in the 200–550 nm range, compared with the smoother absorption intensity of Cu@3D-GCM over the entire wavelength range. This is due to the large size of MNPs, which suggests the efficient use of solar energy.

Similar to the photocatalytic degradation of Cu/Pd@3D-GCM, a UV light irradiation source was applied on three membranes loaded with different types of metal atoms in the presence of H_2_O_2_ after the dark period of RhB adsorption. Figure 3c represents the amount of photocatalytically removed pollutant versus the total amount fed into the reactor. Cu/Pd@3D-GCM showed the highest photocatalytic activity, and the removal rate of RhB reached 98.4% under 90 min irradiation. According to the pseudo-first-order kinetics results, the surface catalytic reaction rate constant (*k*) of the Cu/Pd@3D-GCM sample was calculated to be 0.0459 min^−1^, suggesting impressive catalytic performance (Fig. 3d). In addition, the existence of the porous structures allows light waves to penetrate deeply inside the photocatalyst and leads to more catalytic active sites, thereby enhancing the photocatalytic activity. The hydrogen evolution from the water under visible light irradiation was carried out by using ethanol as the sacrificial electron donor. Typically, Cu/Pd@3D-GCM gave an H_2_ production activity of 1.3474 mmol g^−1^ h^−1^, which is about 7.0-fold (0.1927 mmol g^−1^ h^−1^) higher than that of the Cu@3D-GCM (Fig. 3e). In Table S4, we compare the photodegradation and photocatalytic H_2_ evolution performance of typical graphene-based catalysts. Our approach demonstrates several apparent advantages, including high hydrogen evolution performance, large degradation rate, and easy membrane regeneration. The high photocatalytic activity of Cu/Pd@3D-GCM is attributed to stronger electron affinity of Pd MNPs and the lower hydrogen overvoltage of Pd, making electron transfer from Cu to Pd possible to suppress charge reorganization [41].

### Photocatalytic Mechanism of 3D-GCM in Cleaning Water and H_2_ Production

**Fig. 4:**
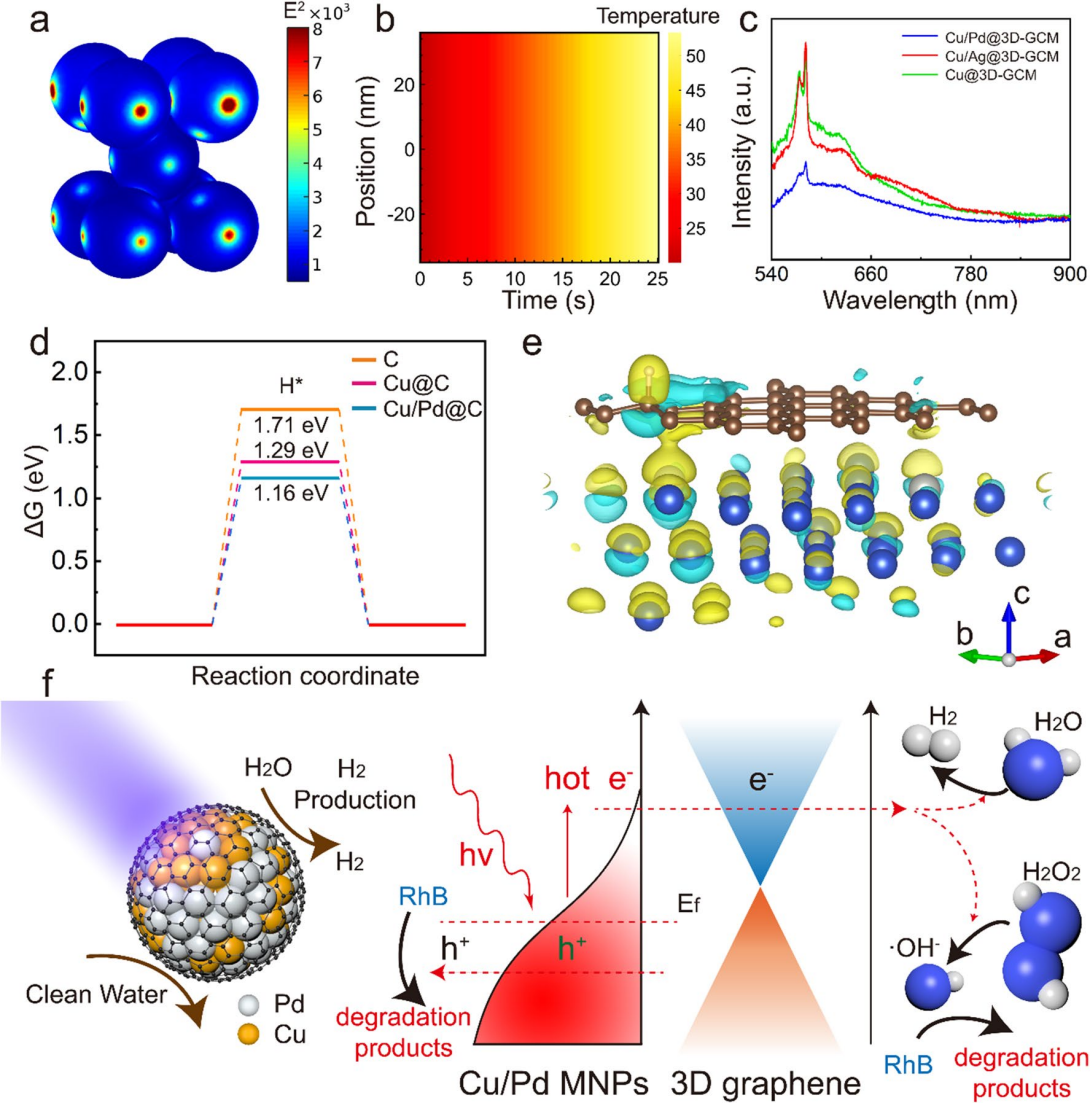
**a, b** Simulated electric field distributions and temperature variation of the sample under light irradiation of 350 nm by three-dimensional finite element analysis. **c** PL spectra of 3D-GCM. **d** The calculated free-energy diagram of the HER on C, Cu@C, and Cu/Pd@C; **e** the charge density redistributions of a 3D-GCM system (The blue, brown, and white atoms represent Cu, C, and Pd, respectively). Blue represents the loss of charge, and yellow represents the gain of electrons in charge density isosurface plot. **f** A schematic diagram of hot-electron generation. The graphene layer is used as a high-efficiency electron acceptor to degrade pollutants and release hydrogen

It is well known that the generated strong electric fields can be non-radiatively damped through generating hot electron–hole pairs via Landau damping [42]. Furthermore, high-concentration hot electrons generated at the surface of the MNPs can interact with phonons to increase the lattice temperature to catalyze chemical reactions in a few hundred femtoseconds [43]. In order to reveal the underlying mechanism, a 3D finite element analysis method was conducted to calculate the intensity of electric near-field and temperature change of the catalytic membrane. In Fig. 4, the color scale bar represents the normalized electric field intensity and temperature (see Supporting Information for the details of the simulation methods). Figures 4a and b and S10 show the electric field distributions and sample temperature variations under 350 nm light irradiation. The enhancement of electric field intensity (*|E|*^*2*^) was clearly observed at the surface of MNPs. In addition, according to the light absorption or PL spectra, we also added the simulation results under other different wavelengths (375, 420, 500, and 750 nm), and shown as following (Fig. S11). The electric field strength distribution of the cross section and the enhancement of the electric field strength value along the central axis also showed fairly strong electric field localization due to its plasmon resonance. Simulation results also indicated an increase in the sample temperature for a short time. The excitation of LSPR greatly enhanced the absorption of light to generate more hot electrons and the electrons tend to be more energetic with the temperature rise. This plays a vital role in activating chemical bonds in chemical transformations.

Based on the above results, the generated electrons need to be rapidly transferred to graphene with high mobility to facilitate hot-electron charge separation from the surface of the nanoparticle, in order to increase the charge lifetime. In addition, the different hydrogen overvoltage between Cu-Pd or Cu-Ag would cause inter-metal electron transfer, further suppressing the charge recombination [41, 42]. To further verify the conjecture, the photoluminescence (PL) emission spectra of the three photocatalytic membranes were measured (the excitation wavelength of 532 nm). As shown in Fig. 4c, Cu/Pd@3D-GCM shows a significantly decreased PL intensity compared with others, demonstrating that the Cu/Pd@3D-GCM sample inhibited the photogenerated carrier recombination. These results verify that the charge transfer of Cu/Pd@3D-GCM could effectively accelerate the separation and transmission of the photoexcited electrons and holes, thereby inhibiting their recombination. Hence, the photocatalytic performance is improved, which is consistent with the results of the H_2_ generation experiment.

As discussed above, 3D-GCM is highly efficient toward hydrogen evolution reaction (HER). Density functional theory calculations were conducted to examine the synergistic performance in 3D-GCM. From the calculation, the adsorption Gibbs free energies (*∆G*) of the adsorbed intermediate H* and charge density distributions of several models including graphene (C atomic) and MNPs encapsulated by graphene (Cu@C, Cu/Pd@C) during the HER process were systematically estimated (* represents an adsorption site) (see Supporting Information for the details of the methods). Figure 4d illustrates the *∆G* values for HER on three samples. The bare graphene had weak H* adsorption and low activity to HER. However, upon encapsulating Cu within graphene, the *∆G* value was effectively increased to yield better HER performance. Furthermore, according to the *∆G* variation, the appropriate Pd doping also effectively improves the HER activity. Thus, Cu/Pd@C shows the best HER activity among the three samples according to the Gibbs free energy calculation.

To further clarify the enhancement of catalytic performance exhibited by the samples after MNPs doping, Fig. 4e shows the charge redistributions in Cu-C, Cu/Pd–C systems, respectively. Specifically, the contact of Cu-C leads to a charge transfer between Cu and C. Furthermore, the appropriate Pd doping rebalances the charge density of the system and further improves the charge transfer efficiency. Therefore, the combination of Cu/Pd and C achieves optimal catalytic performance. The results show that the active sites for the sewage degradation and H_2_ generation are mainly located in metal nanoparticles. In order to compare the effect on reactions under active surface of MNPs and remarkable loss of active surface of MNPs, we supplemented the control experiment to show the effect on reactions under the remarkable loss of active surface of MNPs (Fig. S12). According to the results, the photodegradation efficiency and H_2_ evolution of 3D-Graphene are much lower than those of Cu/Pd@3D-GCM. The weak catalytic performance of 3D-Graphene is attributed to the presence of a small amount of graphene oxide, which is caused by incomplete reduction in laser scribing.

On the basis of previous works and the results above, we propose the following mechanisms of hot-electron generation as shown in Fig. 4f. Upon light illumination, the excited LSPR energy of MNPs tends to yield energetic charge carriers (hot electrons and holes) during the LSPR-decay process. Due to the high conductivity of graphene, electrons could be allowed to be efficiently transferred from MNPs to graphene. The electrons on graphene can reduce the surface H_2_O_2_ and generate a large amount of ·OH and ·O_2_^−^ radicals to degrade organic pollutant molecules. During the hydrogen production process, electrons transferred to graphene exhibit strong reducibility, and H^+^ can be easily reduced into H_2_, as confirmed by the results of the H_2_ generation experiment. Meanwhile, the calculation results confirmed that Pd MNPs can significantly enhanced the performance of photocatalysis, which is attributed to the strong electron affinity of Pd MNPs. In short, H_2_ evolution over Pd is easier than that over Cu and Ag, resulting in the enhancement of photoactivity of the Cu/Pd@3D-GCM.

## Conclusion

In summary, we developed a green, efficient, and universal strategy to fabricate a novel 3D catalytic membrane microreactor. The 3D catalytic membrane microreactor embedded with ultrafine MNPs was engineered from a MOF precursor by laser scribing. Compared with traditional GO microreactors and solvent-based catalysts, our catalytic membrane exhibited superior removal efficiency at ultra-low energy consumption and favorable robustness for aqueous pollutant degradation. The catalytic membrane also showed excellent performance for hydrogen evolution. The superior performance in pollutant degradation and hydrogen evolution of the catalytic membrane was contributed to the synergistic mechanism of Cu, Ag, Pd, and graphene layers that was explored systematically. This work enriches the bimetallic/graphene catalyst system and provides new insights into the catalytic mechanism. Also, the preparation route of large-area 3D-GCM composites provide an efficient and low-cost strategy for addressing environmental concerns and supplying renewable energy.

## Supplementary Information

Below is the link to the electronic supplementary material.

Supplementary file1 (PDF 1953 kb)
