# Supplementary material for: MOF-Like 3D Graphene-Based Catalytic Membrane Fabricated by One-Step Laser Scribing for Robust Water Purification and Green Energy Production
Source: Nanomicro Lett. 2022 Aug 23;14:174. doi: 10.1007/s40820-022-00923-4 (PMC9399326; doi:10.1007/s40820-022-00923-4)
Supplement: Supplementary file 1 — Supplementary file1 (PDF 1953 kb) [file 40820_2022_923_MOESM1_ESM.pdf]

Supporting Information for

MOF-Like 3D Graphene-Based Catalytic Membrane Fabricated by One Step Laser Scribing for Robust Water Purification and Green Energy Production

Xinyu Huang[1, 2, #], Liheng Li[1, #], Shuaifei Zhao[3, #], Lei Tong[1], Zheng Li[1], Zhuiri Peng[1], Runfeng Lin[1], Li Zhou[4], Chang Peng[5], Kan-Hao Xue[1], Lijuan Chen[6], Gary J. Cheng[7, *], Zhu Xiong[3, 8, *], Lei Ye[1, 2, *]

[1]School of Optical and Electronic Information and Wuhan National Laboratory for Optoelectronics, Huazhong University of Science and Technology, Wuhan 430074, P. R. China

[2]Hubei Yangtze Memory Laboratories, Wuhan 430205, P. R. China

[3]Deakin University, Geelong, Institute for Frontier Materials, VIC 3216, Australia

[4]Key Laboratory of New Processing Technology for Nonferrous Metal and Materials (Ministry of Education), Guangxi Key Laboratory of Optical and Electronic Materials and Devices, College of Materials Science and Engineering, Guilin University of Technology, Guilin 541004, P. R. China

[5]College of Chemistry and Materials Science, Hunan Agricultural University, Hunan 410128, P. R. China

[6]School of Material Science and Engineering, Hunan University of Science and Technology, Xiangtan, Hunan Province, P. R. China

[7]School of Industrial Engineering and Birck Nanotechnology Centre, Purdue University, West Lafayette, Indiana, 47907, USA

[8]Institute of Environmental Research at Greater Bay; Key Laboratory for Water Quality and Conservation of the Pearl River Delta, Ministry of Education, Guangzhou University, Guangzhou, Guangdong 510006, P. R. China

[#]Xinyu Huang, Liheng Li and Shuaifei Zhao contributed equally to this work.

*Corresponding authors. E-mail: leiye@hust.edu.cn (Lei Ye); xiongzhu@gzhu.edu.cn (Zhu Xiong); gjcheng@purdue.edu (Gary J. Cheng)

S1 Laser Scribing Mechanism

The transformation of MOF precursors to 3D-GCM is a photothermal process. Upon laser irradiation, the MOF were instantly pyrolyzed by pyrolysis and form a 3D graphene. Simultaneously, due to the high-power intensity of the short pulse laser and the constraints of the slide, high local temperatures up to 2200 K can be achieved during the laser scribing, forming a reducing atmosphere to prevent the oxidation of MNPs. Finally, 3D graphene loaded with metal nanoparticles is realized by laser scribing.

S2 Characterizations of Cu/Pd@3D-GCM

The Brunauer-Emmett-Teller (BET) data of Cu/Pd@3D-GCM is shown in Fig. S2a, which confirms the surface area of the materials. The BET surface area reveals the high surface area of the 3D-GCM after laser scribing. The XPS spectra of Cu/Pd@3D-GCM shows a predominant peak of C 1s at 284.3 eV and O 1s at 530.9 eV (Fig. S2b, c). The FTIR spectrum of the Cu/Pd@3D-GCM is shown in Fig. S2d. The intensity of the absorption peaks at

approximately 3000-3400 cm^{-1}, can be attributed to the O-H bond stretching of surface-active carbon. The peaks at 1645, 1639, and 1480 cm^{-1} corresponding to the C=O symmetric and asymmetric modes, are greatly reduced in the spectra of 3D-GCM. These results can be ascribed to both reductions of GO to graphene and deposition of MNPs. The high-resolution transmission electron microscopy (HRTEM) and Energy-dispersive X-ray Spectrometry (EDS) mapping results are shown in Fig. S3. The uniform elemental distribution of MNPs and C indicates the uniformity and density of the MNPs.

Rhodamine B (RhB) as the contaminant model was filtered from the membranes at 0.1 bar and the permeated solution was collected after filtration to measure contaminant concentrations and recorded the corresponding fluid volumes of permeation. Figure S4 presents the RhB removal efficiency and flux of the Cu/Pd@3D-GCM. In addition, H_2O_2 and UV light was used for the catalytic degradation of pollutants. The separation ability could recover almost 100% of the initial value after 5 cycles of testing.

S3 HRTEM Images and XPS Spectrum of Cu@3D-GCM and Cu/Ag@3D-GCM

Cu@3D-GCM and Cu/Ag@3D-GCM were also prepared using the laser scribing. The TEM image and XPS spectrum are shown in Fig. S7-9. Similar to the Cu/Pd@3D-GCM, these samples show a sheet-like or graphene-like structure, where Cu and Cu/Ag nanoparticles distribute in 3D-graphene homogeneously. The HRTEM image further shows that the lattice fringes of Cu(111) and Ag(111) are observed to be 0.205 nm and 0.235 nm in the amplified TEM photograph, respectively. The results clearly confirm the successful anchoring/deposition of MNPs inside the 3D-graphene.

In addition, the XPS spectra for the as-prepared samples shows a predominant peak of C 1s at 284.3 eV and O 1s at 530.9 eV (Fig. S8-9). The main peaks at 932.6 and 952.08 eV have two shakeup peaks at about 941.375 and 962.375 eV, which proves the presence of Cu^{2+}. Furthermore, the high-resolution XPS spectrum of Cu and Ag for Cu/Ag@3D-GCM shows the Cu 2p and Ag 3d states, respectively. The results show that MNPs were successfully synthesized by laser scribing.

S4 Simulation of Electromagnetic Field Distribution and Temperature Distribution

In order to further depict the localized surface plasmonic resonance (LSPR), the optical spectra and temperature distribution of copper nanoparticles anchored on graphene were simulated with finite element methods using a photothermal-coupling module.

S4.1 Electromagnetic Field Distribution and Absorption Simulation

The modeled structures are illustrated in Fig.S10a. The computational domain is a cubic volume whose edge length is set to 4 times the diameter of the particles. The spherical particles are placed in a body-centered cubic arrangement. A single-layer graphene sheet, which is treated as a conductivity surface in the modeling, is then placed on the surface of the particles. The dielectric function of Cu is taken from the Handbook of Optical Constants of Solids. The dielectric function of graphene is taken from the Drude model [S1].

The dynamic in-plane conductivity (ω) of graphene is derived from the Kubo formula with both intraband and interband contributions. Assuming $k_B T \ll |E_f|$ and $\hbar\omega$, the intraband contribution can be simplified into the Drude-like form [S2-S4].

$$\sigma_{intra} = \frac{2e^2 k_B T}{\pi \hbar^2} ln\left[2Cosh\left(\frac{E_f}{2k_B T}\right)\right]\frac{i}{\omega + i\gamma} \tag{S1}$$

The interband conductivity can be approximated as

$$\sigma_{inter} = -i\frac{e^2}{4\hbar^2} ln\left[\frac{2\left|E_f\right| - \left(\omega - i\gamma^{-1}\right)\hbar}{2\left|E_f\right| + \left(\omega - i\gamma^{-1}\right)\hbar}\right] \tag{S2}$$

Where ω is the angular frequency, e is the charge of an electron, $\hbar = h/2\pi$ is the reduced Planck constant, k_B is Boltzmann's constant, T is the temperature, E_f is Fermi energy of graphene, and γ is the carrier relaxation time.

According to the intraband and interband contributions, the complex permittivity of graphene (ω) is obtained from the complex conductivity:

$$\sigma = \sigma_{intra} + \sigma_{inter} \tag{S3}$$

$$\varepsilon(\omega) = \varepsilon + \frac{i\sigma}{\omega \varepsilon_0 t_g} \tag{S4}$$

Where ε is the out-of-plane component of the effective dielectric permittivity tensor set equal to 1, ε_0 is the permittivity of free space, and t_g is the thickness of graphene.

To simplify calculations, floquet-periodic boundary conditions were used on four sides of the unit cell to simulate the infinite 2D array. The perfectly matched layers (PMLs) on the top and bottom of the unit cell absorb the excited mode from the source port and any higher-order modes generated by the periodic structure. The computational domain was discretized by a structured mesh. The simulation of the field distribution and at and around the Cu nanospheres was carried out with a rigorous Maxwell's solver based on the finite element methods. The implementation of the method dealt with a large number of degrees of freedom needed for the simulation of multiscale structures involving plasmonic effects. The electromagnetic field distribution (Fig. S10) of Cu at 350 nm was obtained by a fit to the Drude model of the empirical data.

S4.2 Temperature Simulation

The temperature distribution was simulated by the steady-state 3D axisymmetric Heat Transfer module in the Solids model. The incident light was absorbed in the copper nanoparticles anchored on graphene, which was modeled as the surface heat source with a boundary temperature of 293.15 K. As shown in Fig. S8, the heating area of nanoparticles contains the entire cube area and the temperature rises rapidly with time.

S5 Calculation Details

To further study the effect of different compositions on HER production, density functional theory (DFT) [S5] calculations for hydrogen adsorption energy were implemented by employing Vienna Ab initio Simulation Package (VASP) code [S6, S7], which is based on the projector augmented-wave technique [S8, S9]. A Pd-doped Cu alloy supercell surface covered by a layer of graphene was used as the basic model, which includes 48 metal atoms and 32 carbon atoms. Moreover, the vacuum layer thickness along the c axis was no less than 1.5 nm for any model supercell. For all calculations, the exchange-correlation energy was treated using the generalized gradient approximation (GGA) within the Perdew-Burke-Ernzerhof (PBE) [S10] functional form. The plane wave energy cutoff was fixed to be 500 eV. Owing to the existence of van der Waals interactions, the DFT-D2 correction was included in our calculations. The convergence criterion for total energy was set to 1.0×10^{-5}eV. The force convergence

criterion was set to 0.02 eV Å^{-1} in each direction. The Brillouin zones for surface models were sampled by 3×3×1 equal-spacing Monkhorst-Pack k-point mesh.

The adsorption Gibbs free energy of intermediate hydrogen (ΔG_{H^*}) on the catalyst is the descriptor for evaluating the HER performance. The adsorption energy is calculated as $\Delta G_{H^*} = \Delta E_{H^*} + \Delta ZPE - T\Delta S$, where ΔE is the adsorption energy of the corresponding intermediate species, ΔZPE is the zero-point energy variation after adsorption, ΔS is the entropy change after adsorption.

S6 Adsorption Kinetics

Adsorption kinetics has been widely studied to describe the adsorption process and mechanism. Kinetics analyses are conducted by using the pseudo-first-order, pseudo-second-order model, which are applied to fit experimental data obtained from experiments [S11, S12]. The equation of pseudo-first-order and the pseudo-second-order kinetic model could be expressed as follows:

$$\frac{dq_t}{dt} = k_1(q_e - q_t) \tag{S5}$$

$$\frac{dq_t}{dt} = k_2(q_e - q_t)^2 \tag{S6}$$

Linear transformations of pseudo-first-order and pseudo-second-order kinetic models are, respectively:

$$Log(q_e - q_t) = Logq_e - \frac{k_1}{e}t \tag{S7}$$

$$\frac{t}{q_t} = \frac{1}{k_2 q_e^2} + \frac{1}{q_e}t \tag{S8}$$

Where t, q_e, and q_t represent time, the adsorption capability at equilibrium and at a certain time t, respectively. k_1 and k_2 are the rate constant of pseudo-first-order and pseudo-second-order model, respectively. The values of q_e, k_1, and k_2 are calculated from the slopes of their respective graphs.

S7 Supplementary Figures and Tables

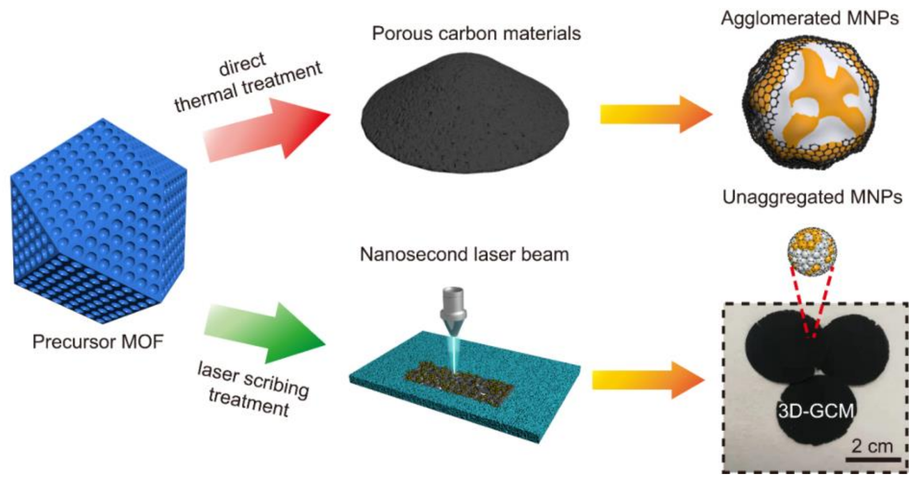

Fig. S1 The process for the preparation of metal-loaded 3D-GCM by laser scribing

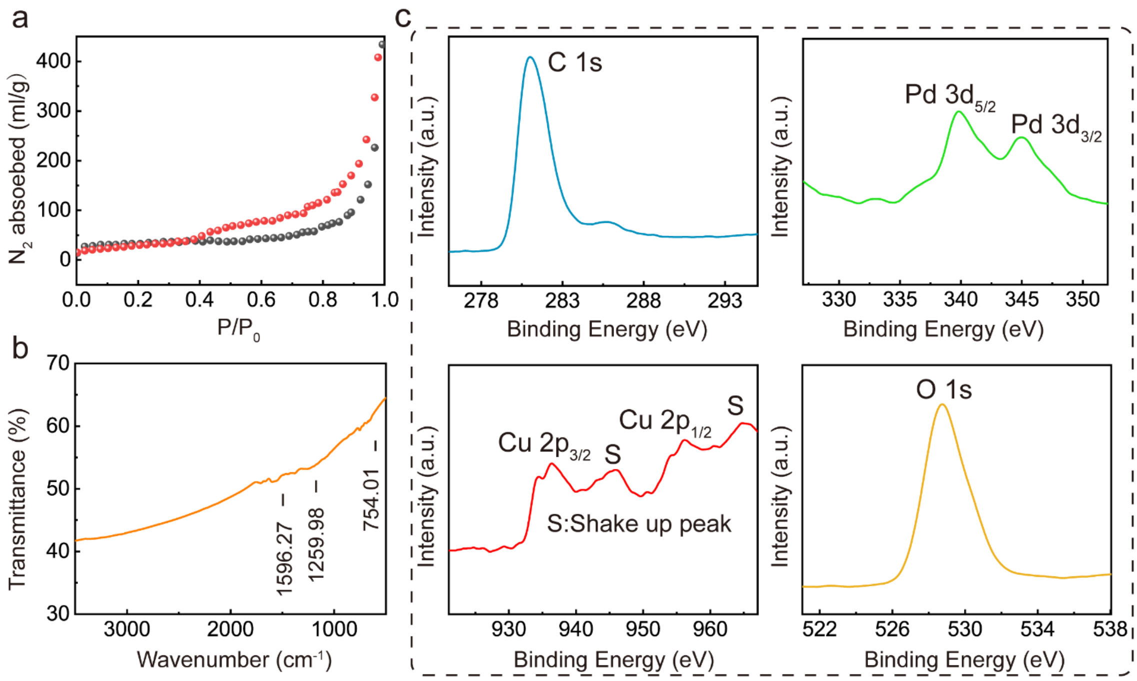

Fig. S2 a N_2 adsorption/desorption isotherm. **b** FTIR spectra of Cu/Pd@3D-GCM. **c** XPS spectra of Cu/Pd@3D-GCM

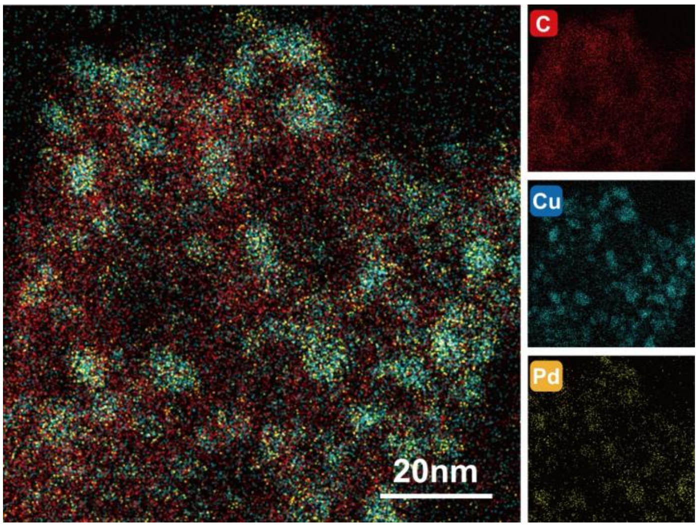

Fig. S3 EDS mappingsand corresponding EDS mappings of C, Cu, Pd of Cu/Pd@3D-GCM

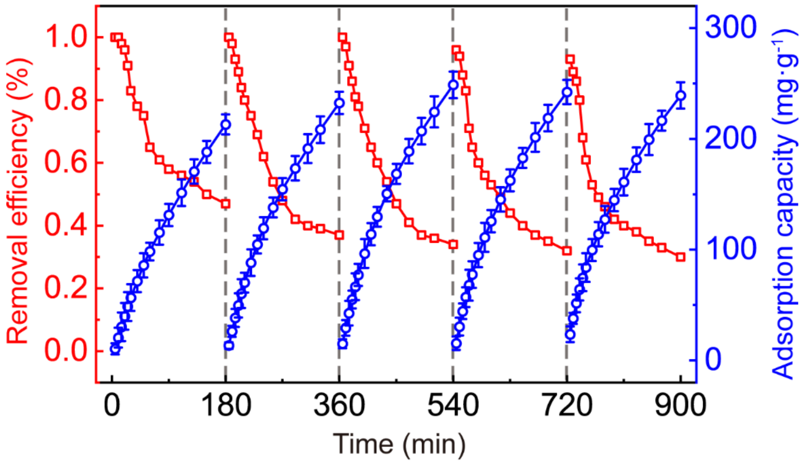

Fig. S4 Removal efficiency and adsorption capacity for RhB of Cu/Pd@3D-GCM during 3h. The removal efficiency could recover almost 100% of the initial value and adsorption capacity increased slightly after 5 cycles of testing

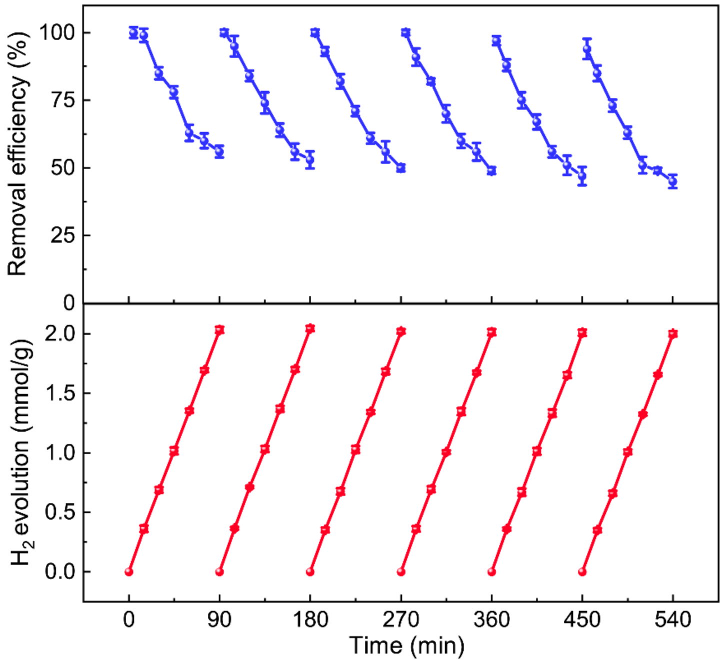

Fig. S5 Five times recycling for removal efficiency and H_2 evolution of Cu/Pd@3D-GCM during 540 mins

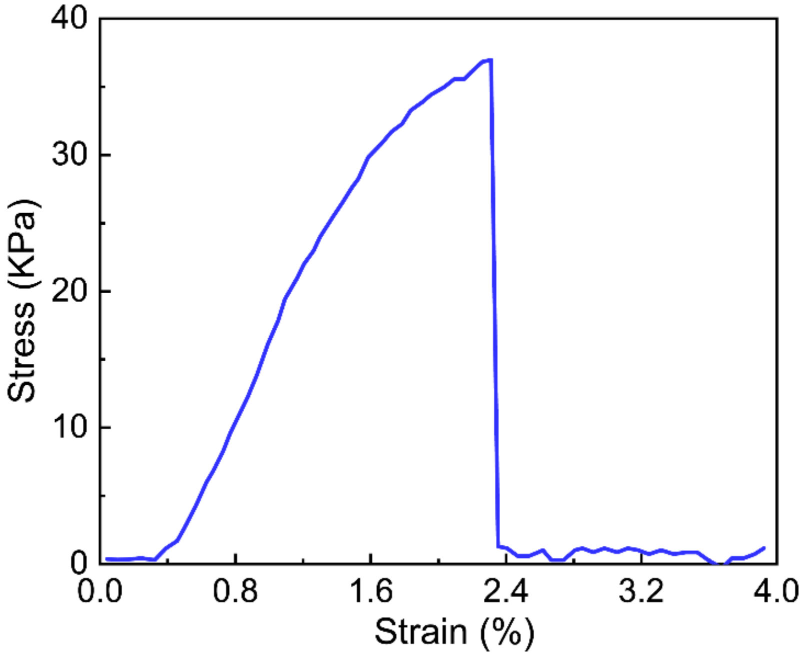

Fig. S6 Mechanical property of Cu/Pd@3D-GCM

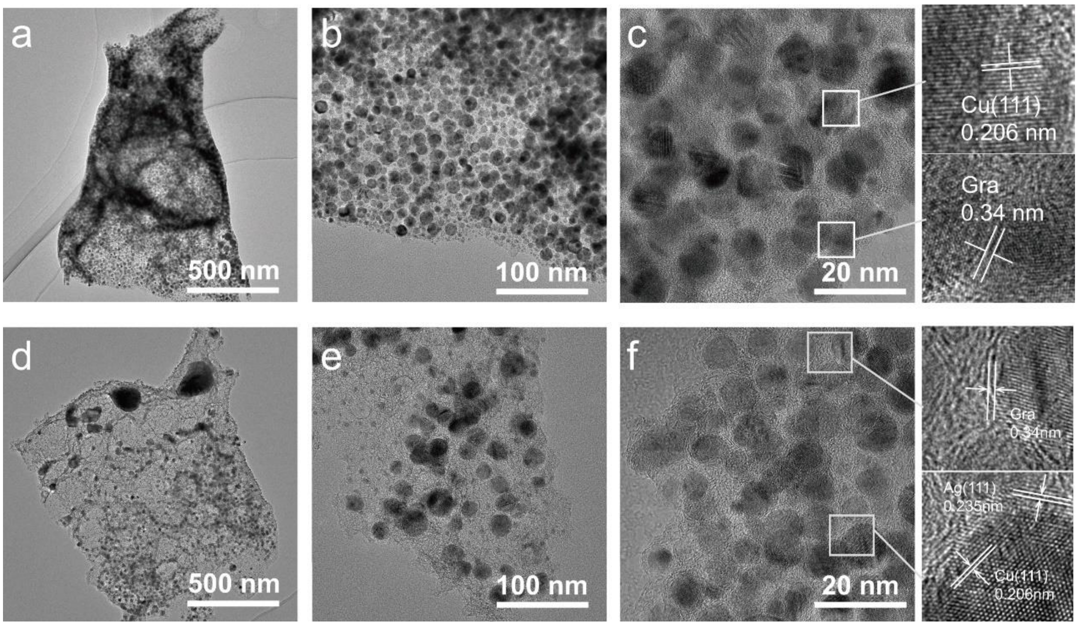

Fig. S7 a-c TEM images, and HRTEM images of Cu@3D-GCM**. d-f** TEM images, and HRTEM images Cu/Ag@3D-GCM

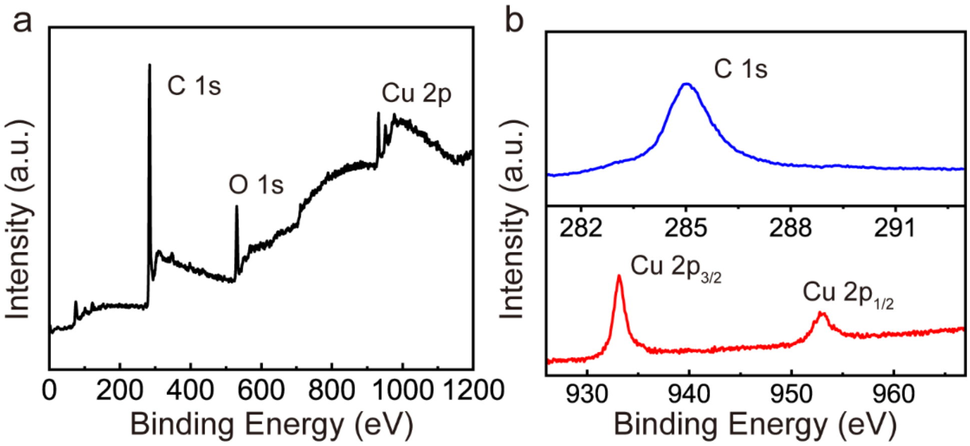

Fig. S8 a XPS spectra of Cu@3D-GCM. **b** High-resolution XPS spectra of Cu@3D-GCM

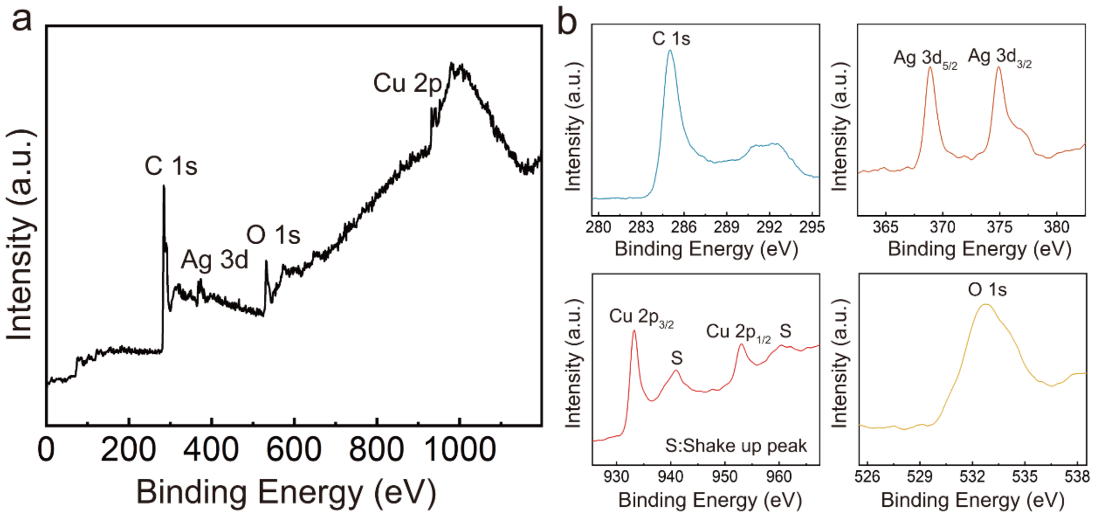

Fig. S9 a XPS spectra of Cu/Ag@3D-GCM. **b** high-resolution XPS spectra of Cu/Ag@3D-GCM

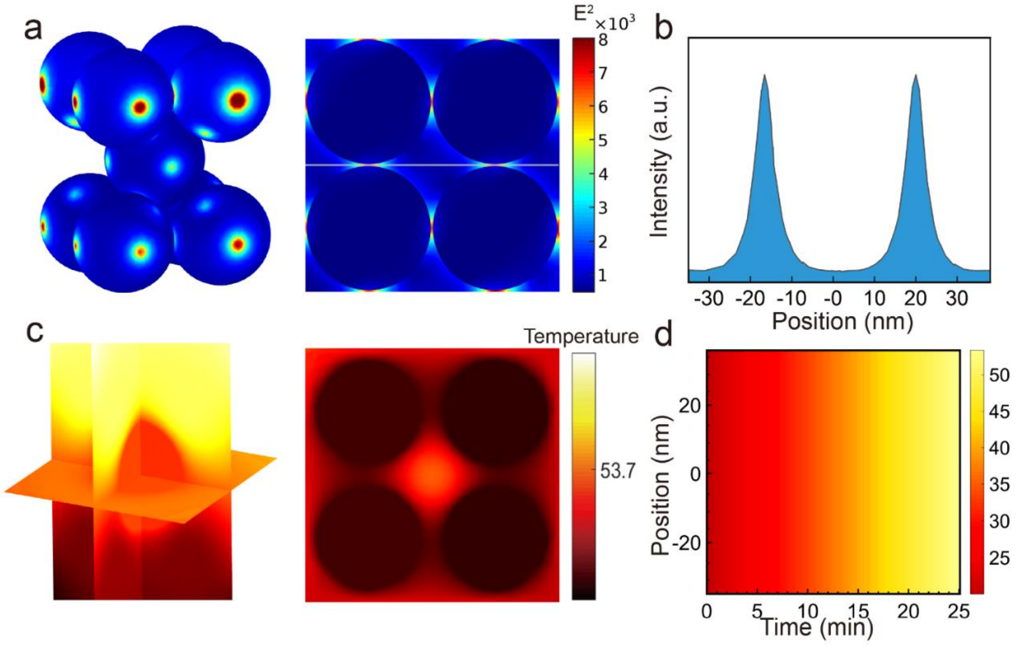

Fig. S10 Spatial distribution of the enhancement of electric field intensity and temperature change at the wavelength of 350 nm of 3D-GCM

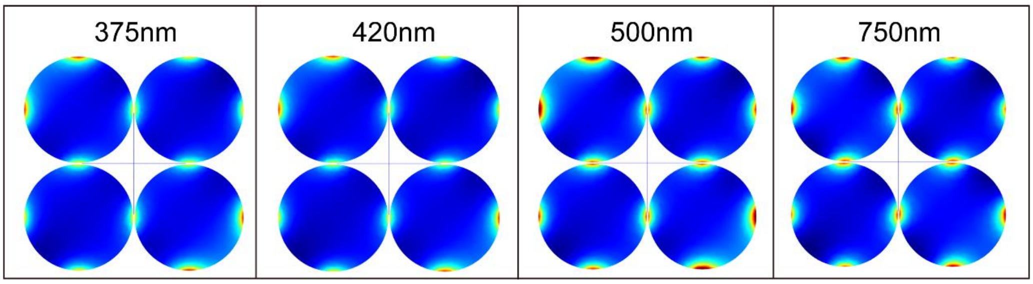

Fig. S11 Spatial distribution of the enhancement of electric field intensity of Cu/Pd@3D-GCM at the wavelength of 375 nm, 420 nm, 500 nm, and 750 nm

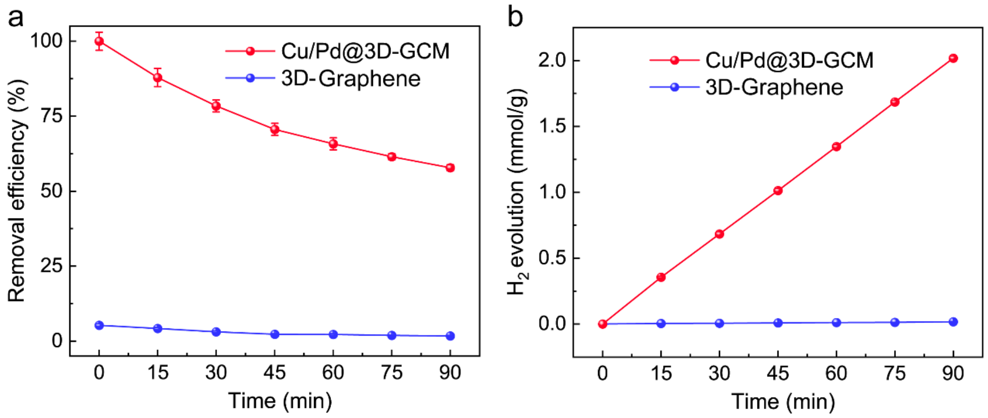

Fig. S12 Removal efficiency and H_2 evolution of Cu/Pd@3D-Graphene and 3D-GCM

Table S1 Fitting data of the pseudo-first-order and pseudo-second-order kinetics model of 3D-GCM for five cycles

Cycles \ RhB	Quasi-first-order dynamics			Quasi-second-order dynamics		
	k_1 ($g \cdot mg^{-1} min^{-1}$)	q_e ($mg \cdot g^{-1}$)	R^2	k_1 ($g \cdot mg^{-1} min^{-1}$)	q_e ($mg \cdot g^{-1}$)	R^2
1	0.0066	299.9698	0.9984	$1.0402*10^{-5}$	452.4887	0.9913
2	0.0093	278.4802	0.9980	$1.6067*10^{-5}$	416.6667	0.9972
3	0.0098	292.4709	0.9983	$1.6606*10^{-5}$	432.9004	0.9982
4	0.0101	279.8460	0.9961	$2.0286*10^{-5}$	396.8254	0.9920
5	0.0103	264.0653	0.9938	$2.2996*10^{-5}$	367.6471	0.9865

Table S2 Fitting data of the pseudo-first-order and pseudo-second-order kinetics models of 3D-GCM for multiple pollutants

Pollutions	Quasi-first-order dynamics			Quasi-second-order dynamics		
	k_1 ($g \cdot mg^{-1} min^{-1}$)	q_e ($mg \cdot g^{-1}$)	R^2	k_1 ($g \cdot mg^{-1} min^{-1}$)	q_e ($mg \cdot g^{-1}$)	R^2
RhB	0.0066	299.9698	0.9984	$1.0402*10^{-5}$	452.4887	0.9913
MB	0.0052	346.1912	0.9994	$5.9103*10^{-6}$	561.7978	0.9955
MO	0.0048	374.8391	0.9990	$6.2096*10^{-6}$	561.7978	0.9857
PNP	0.0086	87.5882	0.9957	$5.6960*10^{-5}$	122.3990	0.9836
AMX	0.0107	63.1786	0.9958	$9.9838*10^{-5}$	88.1834	0.9915
DCP	0.0099	73.5658	0.9990	$6.7387*10^{-5}$	108.5776	0.9989
BPA	0.0096	87.6858	0.9978	$5.6420*10^{-5}$	128.2051	0.9967

Table S3 Comparison of adsorption performance of typical graphene-based catalysts

Adsorbents	q_e (mg/g)	Reference
RGO-MnFe2O4	23.0	[13]
3D RGO-based hydrogels	29.44	[14]
GO-zeolite	55.6	[15]
carboxy-GO/zeolite	67.6	[16]
GO-PDA layer	87.03	[17]
MOF-5@GO	151.5	[18]
In-MOF @GO-2	267.0	[19]
Cu/Pd@3D-GM	299.96	this work

Table S4 Comparison of the photodegradation and photocatalytic H_2 evolution performance of typical graphene-based catalysts

Catalysts	H_2 evolution (umol $h^{-1}g^{-1}$)	Degradation rate constant	Light type	Recyclability (Form)	Reference
CdS - Graphene	1890	——	Visible light (λ > 420 nm)	Difficult (Powder)	[20]
Cu - rGO	59000	——	near-infrared	Difficult (Powder)	[21]
Cu/TiO_2 - graphene	1150	——	Visible light (λ > 420 nm)	Difficult (Powder)	[22]
Cu - graphene	3940	——	Visible light (λ > 420 nm)	Difficult (Powder)	[23]
Au/Pt - rGO	1000	——	Visible light (λ > 420 nm)	Difficult (Powder)	[24]
Au - Graphene	——	0.0025 min^{-1} (Methylene blue)	Visible light (λ > 420 nm)	Difficult (Powder)	[25]
Ag - Graphene	——	0.0029 min^{-1} (Methylene blue) 0.0077 min^{-1} (Congo red)	Visible light (λ > 420 nm)	Difficult (Powder)	[26]
Ag/Au - graphene sheets	——	0.6070 min^{-1} (4-Nitrophenol)	Visible light (λ > 420 nm)	Difficult (Powder)	[27]
TiO_2 - graphene	1260	0.0584min^{-1} (Evans blue dye)	Visible light (λ > 420 nm)	Difficult (Powder)	[28]
CdSe - graphene	335	0.0091 min^{-1} (rhodamine B) 0.0144 min^{-1} (safranine O)	Visible light (λ > 420 nm)	Difficult (Powder)	[29]
ZnO - rGO/C Sponge	14.6	0.0137 min^{-1} (rhodamine B)	Visible light (λ > 420 nm)	Easy (Membrane)	[30]
Cu/Pd@3D-GCM	1374	0.0459 min^{-1} (rhodamine B)	Visible light (λ > 420 nm)	Easy (Membrane)	This work

Supplementary References

[S1] E.D. Palik, *Handbook of Optical Constants of Solids*. (Academic press, 1998).

[S2] Y. Wang, T. Li, S. Zhu, Graphene-based plasmonic modulator on a groove-structured metasurface. Opt. Lett. **42**, 2247 (2017). http://dx.doi.org/10.1364/OL.42.002247

[S3] L.A. Falkovsky, S.S. Pershoguba, Optical far-infrared properties of a graphene monolayer and multilayer. Phys. Rev. B **76**, 153410 (2007). http://dx.doi.org/10.1103/PhysRevB.76.153410

[S4] I. Khromova, A. Andryieuski, A. Lavrinenko, Ultrasensitive terahertz/infrared waveguide modulators based on multilayer graphene metamaterials. Laser Photon. Rev. **8**, 916 (2014). http://dx.doi.org/10.1002/lpor.201400075

[S5] W. Kohn, L.J. Sham, Self-consistent equations including exchange and correlation effects. Phys. Rev. **140**, A1133 (1965). http://dx.doi.org/10.1103/PhysRev.140.A1133

[S6] G. Kresse, J. Furthmüller, Efficiency of ab-initio total energy calculations for metals and semiconductors using a plane-wave basis set. Comput. Mater. Sci. **6**, 15 (1996). https://doi.org/10.1016/0927-0256(96)00008-0

[S7] G. Kresse, J. Furthmüller, Efficient iterative schemes for ab initio total-energy calculations using a plane-wave basis set. Phys. Rev. B **54**, 11169 (1996). http://dx.doi.org/10.1103/PhysRevB.54.11169

[S8] G. Kresse, D. Joubert, From ultrasoft pseudopotentials to the projector augmented-wave method. Phys. Rev. B **59**, 1758 (1999). http://dx.doi.org/10.1103/PhysRevB.59.1758

[S9] P.E. Blochl, Projector augmented-wave method. Phys. Rev. B Condens. Matter **50**, 17953-17979 (1994). http://dx.doi.org/10.1103/physrevb.50.17953

[S10] J.P. Perdew, K. Burke, M. Ernzerhof, Generalized gradient approximation made simple. Phys. Rev. Lett. **77**, 3865 (1996). http://dx.doi.org/10.1103/PhysRevLett.77.3865

[S11] J. Wang, Q. Yao, C. Sheng, C. Jin, Q. Sun, One-step preparation of graphene oxide/cellulose nanofibril hybrid aerogel for adsorptive removal of four kinds of antibiotics. J. Nanomater. **2017**, 5150613 (2017). http://dx.doi.org/10.1155/2017/5150613

[S12] T.A. Tabish, F.A. Memon, D.E. Gomez, D.W. Horsell, S. Zhang, A facile synthesis of porous graphene for efficient water and wastewater treatment. Sci. Rep. **8**, 1817 (2018). http://dx.doi.org/10.1038/s41598-018-19978-8

[S13] S. Bai, X. Shen, X. Zhong, Y. Liu, G. Zhu et al., One-pot solvothermal preparation of magnetic reduced graphene oxide-ferrite hybrids for organic dye removal. Carbon **50**, 2337-2346 (2012). http://dx.doi.org/10.1016/j.carbon.2012.01.057

[S14] J.N. Tiwari, K. Mahesh, N.H. Le, K.C. Kemp, R. Timilsina et al., Reduced graphene oxide-based hydrogels for the efficient capture of dye pollutants from aqueous solutions. Carbon **56**, 173-182 (2013). http://dx.doi.org/10.1016/j.carbon.2013.01.001

[S15] Y. Yu, B.N. Murthy, J.G. Shapter, K.T. Constantopoulos, N.H. Voelcker et al., Benzene carboxylic acid derivatized graphene oxide nanosheets on natural zeolites as effective adsorbents for cationic dye removal. J. Hazard. Mater. **260**, 330-338 (2013). http://dx.doi.org/10.1016/j.jhazmat.2013.05.041

[S16] M. Yusuf, F.M. Elfghi, S.A. Zaidi, E.C. Abdullah, M.A. Khan, Applications of graphene and its derivatives as an adsorbent for heavy metal and dye removal: a systematic and comprehensive overview. RSC Adv. **5**, 50392-50420 (2015). http://dx.doi.org/10.1039/c5ra07223a

[S17] X. Wang, Y. Guo, Z. Jia, H. Ma, C. Liu et al., Fabrication of graphene oxide/polydopamine adsorptive membrane by stepwise in-situ growth for removal of rhodamine B from water. Desalination **516**, 115220 (2021). http://dx.doi.org/10.1016/j.desal.2021.115220

[S18] G. Kumar, D.T. Masram, Sustainable synthesis of MOF-5@GO nanocomposites for efficient removal of rhodamine B from water. ACS Omega **6**(14), 9587-9599 (2021). http://dx.doi.org/10.1021/acsomega.1c00143

[S19] C. Yang, S. Wu, J. Cheng, Y. Chen, Indium-based metal-organic framework/graphite oxide composite as an efficient adsorbent in the adsorption of rhodamine B from aqueous solution. J. Alloys Compd. **687**, 804-812 (2016). http://dx.doi.org/10.1016/j.jallcom.2016.06.173

[S20] Y. Xia, B. Cheng, J. Fan, J. Yu, G. Liu, Unraveling photoexcited charge transfer pathway and process of CdS/graphene nanoribbon composites toward visible-light photocatalytic hydrogen evolution. Small **15**(34), e1902459 (2019). http://dx.doi.org/10.1002/smll.201902459

[S21] P. Zhang, T. Song, T. Wang, H. Zeng, Plasmonic Cu nanoparticle on reduced graphene oxide nanosheet support: an efficient photocatalyst for improvement of near-infrared photocatalytic H_2 evolution. Appl. Catal. B Environ. **225**, 172-179 (2018). http://dx.doi.org/10.1016/j.apcatb.2017.11.076

[S22] Y. Su, S. Li, D. He, D. Yu, F. Liu et al., MOF-derived porous ZnO nanocages/rGO/carbon sponge-based photocatalytic microreactor for efficient degradation of water pollutants and hydrogen evolution. ACS Sustainable Chem. Eng. **6**(9), 11989-11998 (2018). http://dx.doi.org/10.1021/acssuschemeng.8b02287

[S23] T. Huang, Z. Xu, G. Zeng, P. Zhang, T. Song et al., Selective deposition of plasmonic copper on few layers graphene with specific defects for efficiently synchronous photocatalytic hydrogen production. Carbon **143**, 257-267 (2019). http://dx.doi.org/10.1016/j.carbon.2018.11.029

[S24] Z. Lou, M. Fujitsuka, T. Majima, Two-dimensional Au-nanoprism/reduced graphene oxide/pt-nanoframe as plasmonic photocatalysts with multiplasmon modes boosting hot electron transfer for hydrogen generation. J. Phys. Chem. Lett. **8**(4), 844-849 (2017). http://dx.doi.org/10.1021/acs.jpclett.6b03045

[S25] M.E. Khan, M.M. Khan, M.H. Cho, Green synthesis, photocatalytic and photoelectrochemical performance of an Au–graphene nanocomposite. RSC Adv. **5**(34), 26897-26904 (2015). http://dx.doi.org/10.1039/c5ra01864a

[S26] M.E. Khan, M.M. Khan, M.H. Cho, Biogenic synthesis of a Ag–graphene nanocomposite with efficient photocatalytic degradation, electrical conductivity and photoelectrochemical performance. New J. Chem. **39**, 8121-8129 (2015). http://dx.doi.org/10.1039/c5nj01320h

[S27] K. Hareesh, R.P. Joshi, D.V. Sunitha, V.N. Bhoraskar, S.D. Dhole, Anchoring of Ag-Au alloy nanoparticles on reduced graphene oxide sheets for the reduction of 4-nitrophenol. Appl. Surf. Sci. **389**, 1050-1055 (2016). http://dx.doi.org/10.1016/j.apsusc.2016.08.034

[S28] K.Y. Kumar, H. Saini, D. Pandiarajan, M.K. Prashanth, L. Parashuram et al., Controllable synthesis of TiO_2 chemically bonded graphene for photocatalytic hydrogen evolution and dye degradation. Catal. Today **340**, 170 (2020). http://dx.doi.org/10.1016/j.cattod.2018.10.042

[S29] D.C.T. Nguyen, L. Zhu, Q. Zhang, K.Y. Cho, W.C. Oh, A new synergetic mesoporous silica combined to CdSe-graphene nanocomposite for dye degradation and hydrogen evolution in visible light. Mater. Res. Bull. **107**, 14 (2018). http://dx.doi.org/10.1016/j.materresbull.2018.07.006

[S30] M.M. Hasan, S.A. Tolba, N.K. Allam, In situ formation of graphene stabilizes zero-valent copper nanoparticles and significantly enhances the efficiency of photocatalytic water splitting. ACS Sustainable Chem. Eng. **6**(12), 16876-16885 (2018). http://dx.doi.org/10.1021/acssuschemeng.8b04219
